# Supplementary figures and images for: Genome-wide analysis reveals four key transcription factors associated with cadmium stress in creeping bentgrass (Agrostis stolonifera L.)
Source: PeerJ. 2018 Jul 30;6:e5191. doi: 10.7717/peerj.5191 (PMC6071620; doi:10.7717/peerj.5191)

Figure S1 Gene Function Classification (GO)

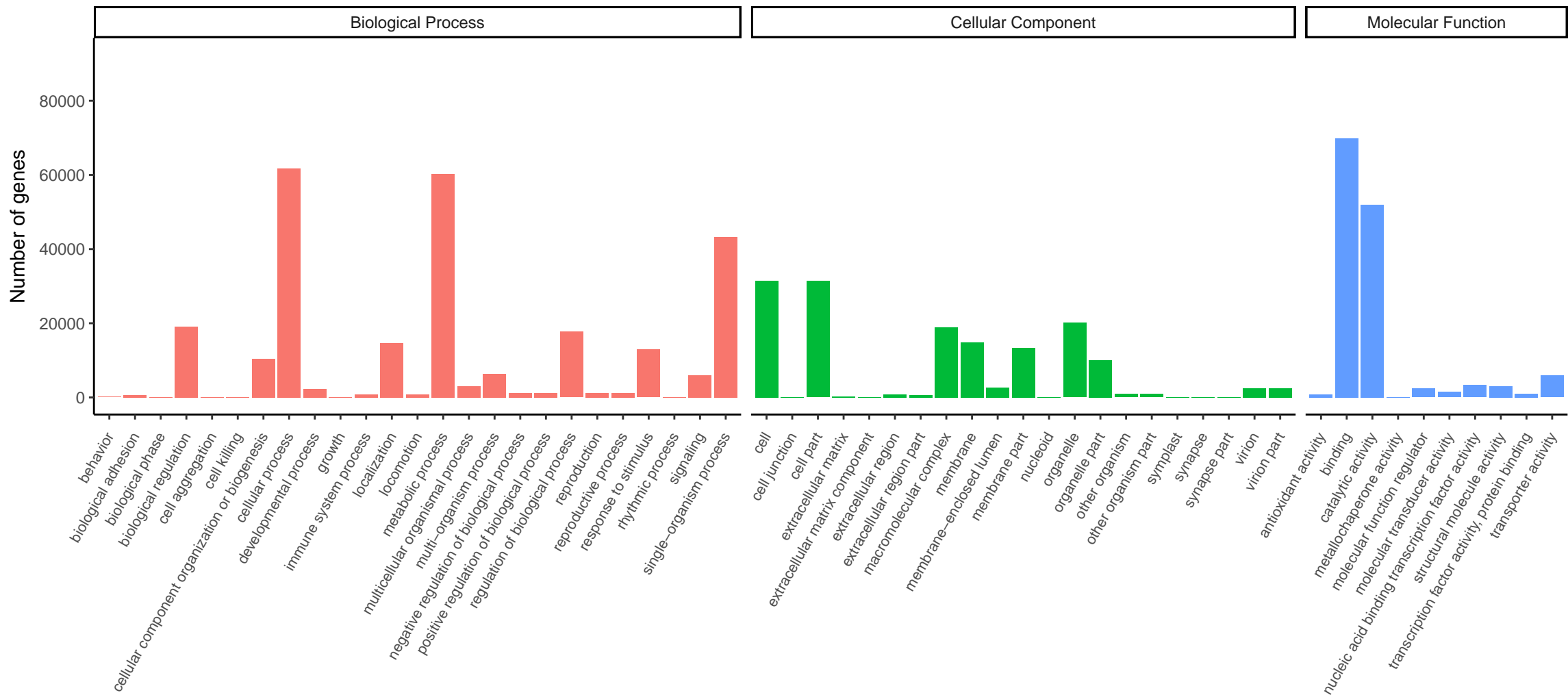

Supplement: Figure S1 [file peerj-06-5191-s001.pdf]
